# Engineering the oleaginous yeast *Yarrowia lipolytica* to produce the aroma compound β-ionone

**DOI:** 10.1186/s12934-018-0984-x

**Published:** 2018-09-01

**Authors:** Jeffrey J. Czajka, Justin A. Nathenson, Veronica T. Benites, Edward E. K. Baidoo, Qianshun Cheng, Yechun Wang, Yinjie J. Tang

**Affiliations:** 1 Department of Energy, Environmental and Chemical Engineering, Washington University, St. Louis, MO 63130 USA; 2 Lawrence Berkeley National Laboratory, Berkeley, CA 94720 USA; 3 Department of Mathematics, Statistics and Computer Science, University of Illinois at Chicago, Chicago, IL 60607 USA; 4 Present Address: Monsanto Company, St Louis, MO 63167 USA; 5 Arch Innotek, LLC, 4320 Forest Park Ave, St. Louis, MO 63108 USA

**Keywords:** ^13^C labeling, Terpenoid, Acetyl-CoA, β-carotene, Machine learning, Fed-batch fermentation, Strain stability

## Abstract

**Background:**

β-Ionone is a fragrant terpenoid that generates a pleasant floral scent and is used in diverse applications as a cosmetic and flavoring ingredient. A growing consumer desire for natural products has increased the market demand for natural β-ionone. To date, chemical extraction from plants remains the main approach for commercial natural β-ionone production. Unfortunately, changing climate and geopolitical issues can cause instability in the β-ionone supply chain. Microbial fermentation using generally recognized as safe (GRAS) yeast offers an alternative method for producing natural β-ionone. *Yarrowia lipolytica* is an attractive host due to its oleaginous nature, established genetic tools, and large intercellular pool size of acetyl-CoA (the terpenoid backbone precursor).

**Results:**

A push–pull strategy via genome engineering was applied to a *Y. lipolytica* PO1f derived strain. Heterologous and native genes in the mevalonate pathway were overexpressed to push production to the terpenoid backbone geranylgeranyl pyrophosphate, while the *carB* and biofunction *carRP* genes from *Mucor circinelloides* were introduced to pull flux towards β-carotene (i.e., ionone precursor). Medium tests combined with machine learning based data analysis and ^13^C metabolite labeling investigated influential nutrients for the β-carotene strain that achieved > 2.5 g/L β-carotene in a rich medium. Further introduction of the carotenoid cleavage dioxygenase 1 (CCD1) from *Osmanthus fragrans* resulted in the β-ionone production. Utilization of in situ dodecane trapping avoided ionone loss from vaporization (with recovery efficiencies of ~ 76%) during fermentation operations, which resulted in titers of 68 mg/L β-ionone in shaking flasks and 380 mg/L in a 2 L fermenter. Both β-carotene medium tests and β-ionone fermentation outcomes indicated the last enzymatic step CCD1 (rather than acetyl-CoA supply) as the key bottleneck.

**Conclusions:**

We engineered a GRAS *Y. lipolytica* platform for sustainable and economical production of the natural aroma β-ionone. Although β-carotene could be produced at high titers by *Y. lipolytica*, the synthesis of β-ionone was relatively poor, possibly due to low CCD1 activity and non-specific CCD1 cleavage of β-carotene. In addition, both β-carotene and β-ionone strains showed decreased performances after successive sub-cultures. For industrial application, β-ionone fermentation efforts should focus on both CCD enzyme engineering and strain stability improvement.

**Electronic supplementary material:**

The online version of this article (10.1186/s12934-018-0984-x) contains supplementary material, which is available to authorized users.

## Background

Terpenoids are a class of secondary metabolites that have a wide-range of biochemical functions in nature, including pigments, anti-oxidants, signaling molecules, and plant defense mechanisms [1–3]. The diverse properties of terpenoids have led to their wide-spread use in the cosmetic, pharmaceutical, and fragrant and flavor industries [4–6]. β-Ionone, an apo-carotenoid (derived from a C40 terpenoid, also known as a carotenoid), is one such molecule. The chemical has an intense, woody smell and a low odor threshold [7] that has led to its utilization in industrial fragrance products, such as perfumes, personal care products, and as a flavoring agent in non-alcoholic beverages and gelatins [8]. The yearly global ionone market averaged approximately $166 million from 2011 to 2015, but demand is expected to increase due to new emerging markets in Brazil, China, and India. In addition to the growing demand in existing market segments, β-ionone has the potential for applications in the health care and pharmaceutical markets. For example, it demonstrated the ability to inhibit breast cancer cells in vivo [9] and to limit tumor incidence in a rat cancer model [10]. Commercial β-ionone can be chemically synthesized, but such a product is less valuable than plant derived β-ionone due to recent increases in consumer desire for natural food products (i.e., natural aroma compounds have a range of 10–100 fold price increase over the chemically synthetic alternative) [11]. Natural aroma molecules can be obtained via extraction from plants [12], but low biochemical abundance makes product isolation labor intensive and costly. Raspberries, one of the most abundant sources of ionone, produce only 1.72 mg of β-ionone/kg of wet weight [7]. Increasing ionone production through growing or obtaining more crop biomass is not a feasible long-term solution due to land scarcity, crop utilization priorities, and unpredictable weather and climate change (which also introduces instability into the current supply chain) [13]. A second method for obtaining natural β-ionone involves using in vitro enzymatic processes (i.e., the biotransformation of β-carotene to β-ionone). However, the enzymatic bio-transformations often lead to a variety of undesirable byproducts [11, 14]. These specific limitations motivate research into new reliable production processes for obtaining natural β-ionone.

**Fig. 1:**
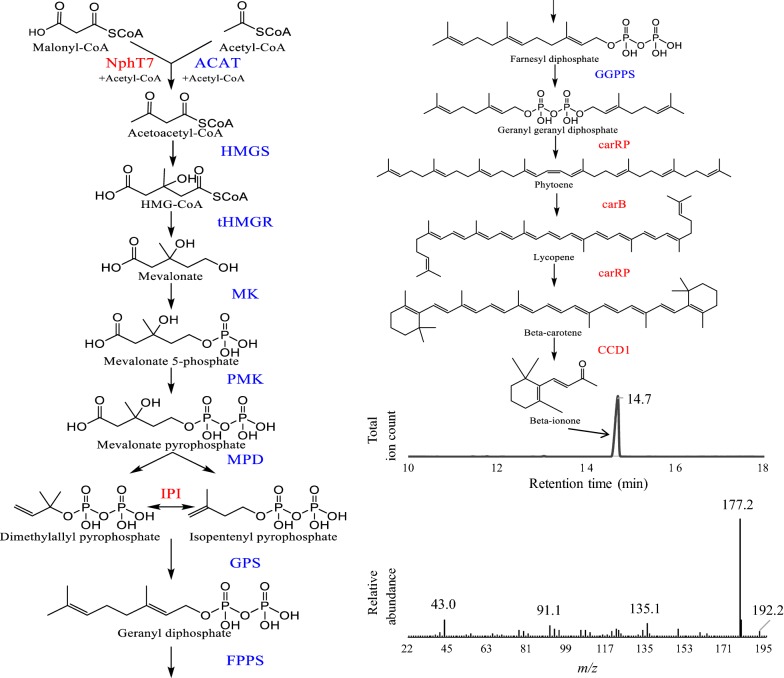
Pathway map for synthesis of β-ionone starting from acetyl-CoA. Enzyme abbreviations are as follows: Acetyl-CoA thiolase (*ACAT*), Acetoacetyl CoA synthase (*NphT7*), HMG-CoA synthase (*HMGS*), Truncated HMG-CoA reductase (*tHMGR*), Mevalonate kinase (*MK*), Phosphomevalonate kinase (*PMK*), Mevalonate pyrophosphate decarboxylase (*MPD*), IPP isomerase (*IPI*), geranyl phosphate synthase (*GPS*) a fusion gene of farnesyl pyrophosphate synthase (FPPS) and geranyl pyrophosphate synthase (GGPPS), a bifunctional lycopene cyclase/phytoene synthase (carRP), phytoene dehydrogenase (carB), and carotenoid cleavage dioxygenase 1 (CCD1). Blue enzymes represent pathway steps overexpressing native *Y. lipolytica* enzymes. Red names represent heterologous enzymes introduced. Spectra for β-ionone gas-chromatography peak (at ~ 14.7 min) (top) and corresponding mass spectra (bottom) analysis of dodecane extract layer. Small α-ionone peaks were observed at ~ 11.8 min. GC–MS data showed the recovered ionone with high purity

Metabolic engineering is a promising alternative approach, as the terpenoid biochemical pathways and genes have been elucidated (Fig. 1) [15]. While much effort has focused on developing microbial platforms for production of terpenoids, few papers have reported aroma production. Recently, *Escherichia coli* was successfully engineered to produce 0.5 g/L of β-ionone [16]. However, the US Food and Drug Administration (FDA) does not categorize *E. coli* as a GRAS microorganism. Efforts in the GRAS yeast *Saccharomyces cerevisiae* have resulted in yields too low for viable industrial production (1 mg/g dry cell weight) [17, 18]. One promising microorganism is the non-conventional yeast *Yarrowia lipolytica*. This GRAS species offers the potential to become a new production platform for β-ionone due to its innate metabolic capabilities [19]. Particularly, *Y. lipolytica* has a rich acetyl-CoA pool due to its cytosolic ATP citrate lyase (ACL) [20, 21]. Genetic tools have been established in *Y. lipolytica* [22], and several groups have successfully engineered the species to produce diverse carotenoids [23–25]. Another industrial advantage of utilizing *Y. lipolytica* is its capacity to grow on a large range of carbon sources (including but not limited to: glucose, glycerol, alcohols, and fatty acids), allowing for the use of diverse and cheap feedstock [26]. In addition, the yeast’s biomass can be incorporated into animal feed, providing a secondary revenue source [27]. Currently, the species is widely used in industry as a single cell protein factory and for production of products such as citric acid and peach flavor [28]. Here, we report engineering *Y. lipolytica* to produce β-ionone via overexpression (Push) of the mevalonate pathway, the introduction of three heterologous enzymes (Pull), and fermentation optimization.

## Results

### Genetic engineering

For carotenoid synthesis, the co-expression of *carB* and *carRP* genes from *Mucor circinelloides* led to production of β-carotene, confirmed by HPLC analysis. The further integration of a carotenoid cleavage dioxygenase 1 of *Osmanthus fragrans* (OfCCD1) into the genome led to detectable production of β-ionone (see Fig. 1 for chromatogram). *Y. lipolytica* contains a native mevalonate (MVA) pathway that operates with relatively low flux [29]. To circumvent the native regulatory network and push flux through the pathway, two heterologous genes (NphT7 from *Streptomyces* sp. [30] and IPI from *Haemotoccus pluvialis* [31, 32]) were integrated into the genome. Native genes in the MVA pathway were also overexpressed by increasing their copies in the genome (Fig. 1), as described in previous papers [29, 33–35]. Each gene was expressed under the same constitutive promoter (TEF) and terminator (XPR2).

### Medium optimization for β-carotene production

**Fig. 2:**
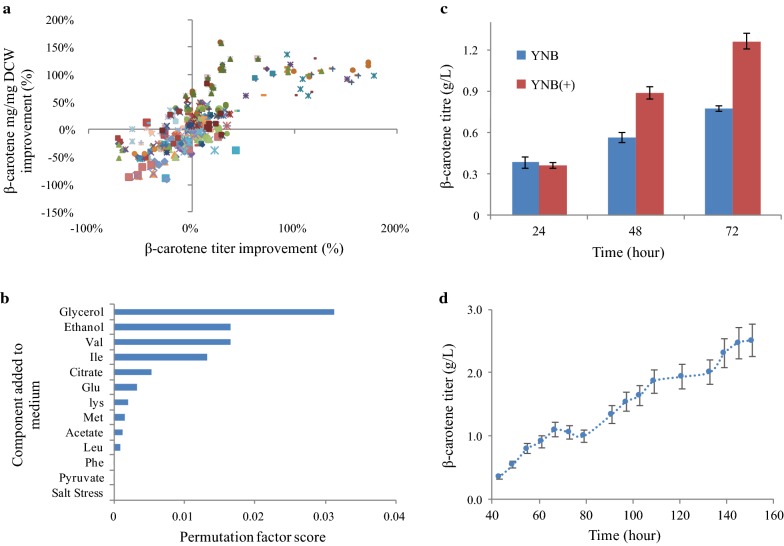
Data and analysis from medium optimization experiments. **a** Spread of data representing the β-carotene titer and yield/DCW percent improvement relative to strains grown in the base (YNB only) medium. Each shape and color represents a separate medium condition (labels not shown due to visual limitations, see Additional file 1); **b** The feature ranking of the medium components on the predictive power of the gradient boosted tree machine learning model; **c** Comparison of the β-carotene titers achieved for the YNB medium and YNB(+) medium in 25 mL shake flask (YNB medium was supplemented with an additional 2 g/L glucose to match the primary carbon source concentrations); **d** β-carotene titers achieved in bench-top fermenter using a YPD medium (error bars represent measurement variations)

β-Ionone (a volatile compound) can be difficult to capture and quantify, while β-carotene is detectable via UV Spectrophotometry [36]. Therefore, we utilized the β-carotene strain as proxy for β-ionone production as a high throughput screening of strain response to medium conditions. Yeast-peptone-dextrose (YPD) medium is used to promote *Y. lipolytica* biosynthesis, but it contains a complex mix of nutrients. To identify key nutrients that specifically contribute to β-carotene production, shaking tube medium tests in the defined Yeast-Nitrogen Base (YNB) medium supplemented with varying amino acids and carbon co-substrates were performed. Specifically, as acetyl-CoA is thought of being the limiting precursor for the MVA pathway, carbon sources that directly contribute to the acetyl-CoA pool (acetate, citrate, ethanol, etc.) were tested. In addition to revealing beneficial medium components, the outcomes could also reveal whether acetyl-CoA supply was rate limiting during *Yarrowia* bio-production. However, the experimental variation in medium components and biological noise made it difficult to determine which factors were beneficial (Fig. 2a, Additional file 1). Machine learning, a powerful tool for analyzing complex data [37, 38], was utilized to decipher the data. The trained model reached 89% prediction accuracy on the test data, while a permutation feature importance score verified the indicators of high production (Fig. 2b). Based on the model analysis, glycerol was the most beneficial component, leading to increased titers of ~ 30%. The addition of excess isoleucine and valine (whose syntheses consume acetyl-CoA) were the next most important factors. Lastly, supplementation of ethanol during the late growth phase was also beneficial.

### β-Carotene production in optimized medium

With information from the high throughput shaking tubes, a YNB(+) medium was developed that augmented YNB medium with glycerol, valine, and isoleucine. Ethanol was also added during the mid-growth phase (see “Methods”). The YNB(+) medium moderately benefited β-carotene production. In shaking flask cultures, an average improvement of 43% ± 7% (p-value < 0.01) in β-carotene titer (1.26 ± 0.05 g/L) was achieved compared to the YNB cultures (Fig. 2c). The cells grown in defined YNB(+) medium showed productivity close to cells grown in YPD medium (rich in yeast and peptone). In a representative fed batch fermentation with a 2 L bioreactor, the cells cultured in YPD medium with glucose supplements achieved a titer close to ~ 2.5 g/L at 160 h (Fig. 2d), similar to previously reported efforts [23].

## ^13^C-Isotopic labeling of central metabolites

**Fig. 3:**
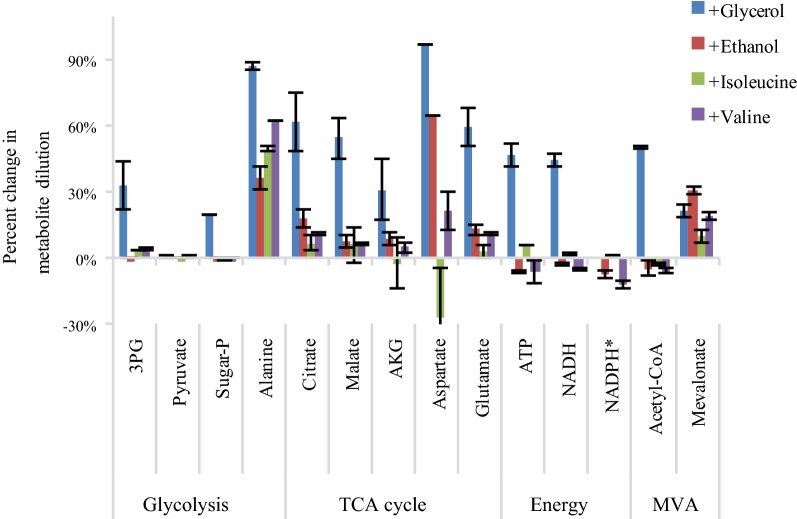
Isotopic labeling results. Bars show the percent change in the metabolite dilution relative to the control (base line medium supplemented with U-^13^C glucose), which is calculated by: Metabolite labeling dilution percentage = (M_control_ − M_test_)/M_control_. M_control_ is Metabolite ^13^C percentage from the fully labeled glucose culture; M_test_ is metabolite ^13^C percentage from tested conditions. Error bars represent the standard deviation (n = 2)

Isotopic tracing experiments were performed to gain molecular insight into the contribution of nutrient supplements to the metabolism of *Y. lipolytica*. Cell cultures were grown in the YNB medium with U-^13^C (fully labeled) glucose as the carbon source. Unlabeled secondary carbon sources or amino acids (glycerol, ethanol, isoleucine, or valine) were pulsed into the YNB cultures during the early exponential phase. The cell metabolism was quickly quenched using liquid nitrogen in the late growth phase, and the metabolites were extracted and analyzed via LC/MS (see “Methods”). The dilution of labeling relative to the fully labeled glucose control experiment was used to assess each component’s contribution (Fig. 3). Overall, glycerol had the largest carbon contribution to the central metabolism, having 30% or more dilution change in most metabolites (including energy molecules ATP and NADH) relative to the control. Ethanol had only minor contributions to the central metabolites; however, it contributed to the mevalonate pool since its degradation promotes cytosolic acetyl-CoA pool (not mitochondrial acetyl-CoA pool). The amino acid supplements led to minor changes in central metabolite labeling (< 10%), indicating sufficient amino acid synthesis capability in *Yarrowia*.

### In situ ionone trapping

**Fig. 4:**
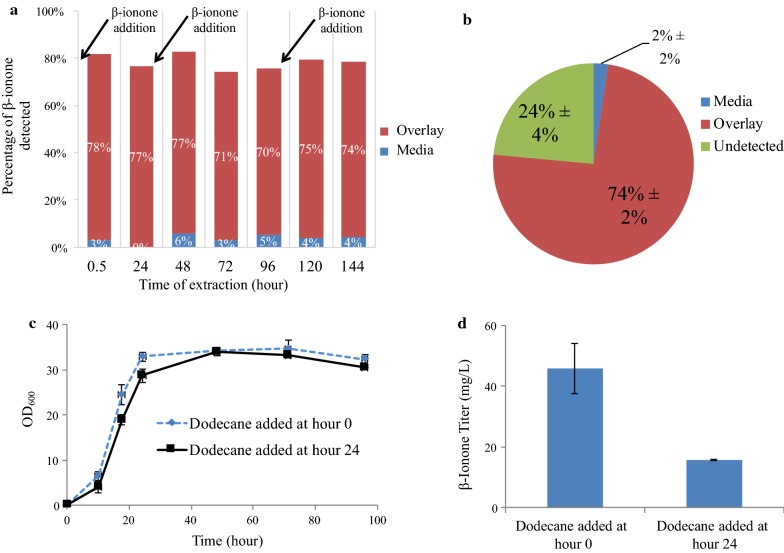
Extraction efficiencies and shaking flask fermentation. **a** Graph showing the relative stability of the extraction method over time; **b** The percentage of β-ionone retained 24 h after a pulse in the 2 L fermenter, with an air flow rate of 0.5 SLPM at a pH 5.5; **c** Growth curves of the β-ionone strain in shaking with a 10% dodecane overlay. The dodecane was either added at immediately (0 h, dashed blue line) or after 24 h (sold black line); **d** Measured β-ionone titers in shaking flask from **c** after 24 h. Error bars represent standard deviation (n = 3 in **a**, 2 in **c** and **d**)

As previously mentioned, β-ionone is a volatile compound with limited aqueous solubility (169 mg/L in water at 25 °C [39]). In a control experiment, 80% of β-ionone (pulsed at relevant production titers of 0.5 and 1 g/L) was lost from the aqueous medium in our fermenter set-up within 1 day. Since β-ionone has been reported to be soluble in alcohol and organic solvents [40], an organic compound was explored as means to capture the product. Dodecane is a relatively non-volatile compound that has previously been used as an organic overlay to trap volatile terpenoids during cell fermentations, including β-ionone [18, 41, 42]. Thus, we investigated the ability of dodecane to partition and trap β-ionone during our bioreactor growths. 0.5 g/L of β-ionone was pulsed at three separate times into the 2 L fermenter containing a 10% by volume dodecane layer and YPD medium to obtain biologically relevant concentrations in the range of 0–1.5 g/L (Fig. 4a). Our analysis determined that dodecane phase captured 74% of β-ionone, while 2% of compound remained in the aqueous phase, leading to an overall capture efficiency of 76% (Fig. 4b). It was found that the dodecane addition did not adversely affect the β-ionone strain’s growth in shaking flask (Fig. 4c) and that the time of addition (0 vs 24 h of growth) did not lead to a significant increase (p value = 0.16) in the titer achieved (Fig. 4d). Therefore, all ionone fermentations were performed with the dodecane present in the medium from the start.

### β-Ionone production in fed-batch fermentations

**Fig. 5:**
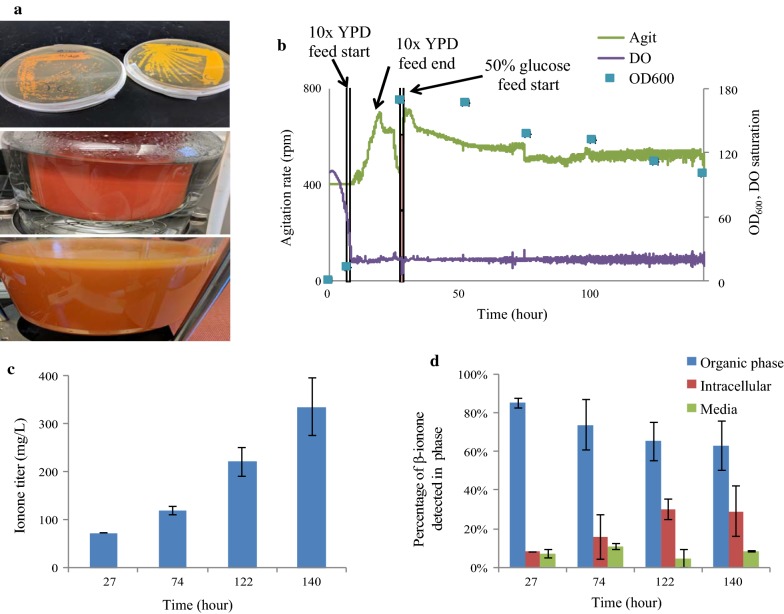
Strain fermentations **a** (Top) Comparison of β**-**carotene strain (left) and β-ionone strain (right) grown on YPD plates, (middle) pigmentation of bioreactor at the end of β-carotene strain fermentation (dark orange color), and (bottom) β-ionone strain fermentation (light orange color); **b** Representative fermentation parameters; **c** β-ionone fermentation profile; **d** Distribution of β-ionone extract from each phase analyzed (intracellular, dodecane overlay, and aqueous medium)

Fermentation of β-ionone strains occurred in a 2 L vessel operating in fed-batch mode for 140 h. The dodecane overlay was kept at 10% of the working volume. β-Ionone was measured in three components; the dodecane overlay, the medium, and the intracellular component. Attempts to separate any volatized β-ionone by bubbling the off-gas through one or two stages of chilled dodecane led to no appreciable absorption (< 1 mg captured). At the end of the fermentation, a highest titer of 380 mg/L was achieved in the glucose fed fermentation (Fig. 5). The co-fed fermentation of additional glycerol or optimized medium components (glycerol, ethanol, isoleucine, and valine) did not obtain higher β-ionone titers vs the glucose fed fermentation. Interestingly, despite the volatility of the compound, a significant portion of the total β-ionone titer was retained intracellularly at 140 h (~ 30%, Fig. 5d). This intracellular portion increased as β-ionone was produced throughout the fermentation. *Y. lipolytica* is known to utilize complex lipid bodies to store lipids and fat soluble compounds, in which the intracellular ionone may have partitioned [43]. The β-ionone strain also showed little accumulation of β-carotene (1.8 ± 0.3 mg/L at 140 h) during the fermentations. Overall, the achieved ionone titer was about tenfolds lower compared to the β-carotene strain’s titers. Rather than low MVA pathway flux or carotene synthesis, the last enzyme OfCCD1 is possibly the key bottleneck due to poor activity or non-specific cleavage of β-carotene.

### Engineered strain stability

**Fig. 6:**
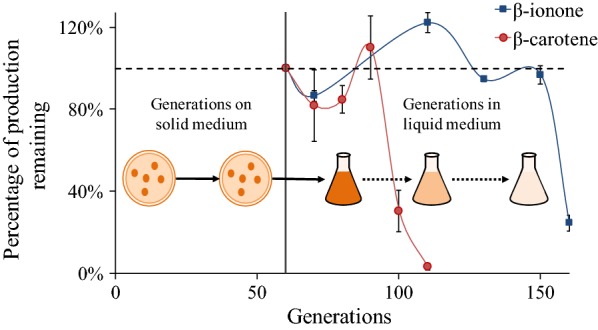
Depiction of strain stability subculture experiments and results versus the number of generations accumulated. There was no selection pressure in the agar plate medium, and we estimated ~ 30 doubling times (i.e., generations) for engineered strain to form colonies from each plating based on the mass of a single cell [54]. The engineered strain was plated twice before liquid subcultures. Solid vertical line indicates transition from solid plate growths to liquid medium

For industrial bio-manufacturing using engineered hosts, strain stability is essential for commercial viability. Here, we tested the production stability of the engineered β-carotene and β-ionone strains by continuously sub-culturing them on YPD plates (twice) and in shake flask cultures (~ 100 doubling times). As shown in Fig. 6, both strains became unstable after extended sub-culturing. The β-ionone strain maintained its production level over more generations than the β-carotene strain, possibly due to its lower productivity and product accumulation (and thus lower metabolic burden and stress).

## Discussion

Metabolic engineering often employs Push (increase precursor supply) and Pull (improve end-product enzyme functions) strategies to enhance productivity. Acetyl-CoA is a key terpenoid precursor needed to push flux through the over-expressed MVA pathway. While *Y. lipolytica* has large quantities of cytosolic acetyl-CoA due to its ACL [20, 21], many important competing pathways, including fatty acids and lipids synthesis, can quickly drain the available resources. Interestingly, the supplementation of citrate and acetate (direct carbon contributors for cytosolic acetyl-CoA) did not show significant improvement in the β-carotene producing strain’s titer (Fig. 2a). Ethanol supplementation did enhance the titer, but isotopic tracing via LC–MS (Fig. 3) only indicated a small contribution to acetyl-CoA pools. Isotopic data indicates that the β-carotene strain had a sufficient cytosolic acetyl-CoA pool for both biomass and product production, allowing for relatively high titers of β-carotene.

Thus, evidence points to the last enzymatic step in β-ionone production, catalyzed by the CCD1 enzyme, as the rate-limiting step (i.e., glycerol supplementation increased β-carotene strain’s titers but not the β-ionone strain’s titers). Similar issues were previously reported with β-ionone engineering efforts in *E. coli*, where the authors discovered the OfCCD1 enzyme had low expression and non-specific cleavages of the precursors phytoene and lycopene [16], which is consistent with poor conversion of β-carotene to ionone in our engineered strain. Increasing the solubility and expression of OfCCD1 through a fusion-protein partner did not lead to a significant increase of β-ionone production by *E. coli* [16]. Furthermore, only slight improvement of production was achieved through introduction of different CCD1 enzymes from either *Vitis vincefera* (VvCCD1) or *Petunia hybrid* (PhCCD1) in *E. coli* [16]. This paper further supports the CCD1 enzyme as a key target for future engineering efforts. Interestingly, the *E. coli* system containing the OfCCD1 enzyme reached a β-ionone titer of 32 mg/L in shaking flask conditions [16]. Our *Y. lipolytica* shaking flask titers nearly doubled the previously obtained titer (60 ± 8 mg/L). However, our larger scale 2 L fermentation did not surpass the *E. coli* titer in a 250 mL mini-bioreactor (380 mg/L vs 500 mg/L), possibly due to higher loss of ionone from bioreactor off gas as well as the instability of carotenoid yeast strain during process scale-up.

Yeast engineering for aroma production is still challenging with many potential bottlenecks. Inefficient enzyme steps often limit productivity while the introduction of long heterologous enzymatic pathways cause metabolic burdens, even with genome integration [44]. β-Ionone production requires further engineering and optimization efforts to improve the rate and specificity of the CCD1 catalyzed enzymatic transformation of β-carotene to β-ionone. In addition, the high levels of carotenoid intermediates may introduce cell stress, leading to strain instability after successive subcultures. One approached aimed at increasing production and minimizing metabolic burden is modular engineering, through which small sub-sections of the pathway are individually optimized [45–47]. This approach can potentially improve β-ionone strain stability for the industrial applications. Finally, the β-ionone extraction efficiency using dodecane may be over-estimated during bioreactor operations (i.e., due to complex interactions among gas/liquid/solid interfaces). Therefore, improving the efficiency of the product extraction and capture is necessary to ensure successful production on an industrial scale. We will continue work on enzyme engineering to address the encountered issues as well as improving the means of product extraction and capture, aiming to achieve the break-even point of 1 g/L for commercial production [14].

## Conclusion

*Yarrowia lipolytica* is a promising industrial microbe that has the potential to be utilized for production of terpenoid compounds. Here, to our knowledge, we have successfully engineered β-ionone production in *Y*. *lipolytica* for the first time. We also identified the last enzymatic step in β-ionone production as a key bottleneck as further engineering target.

## Methods

### Chemicals, medium compositions, and cell growths

Yeast nitrogen base (YNB) and synthetic drop-out complete mix (SD drop-out) were purchased from US Biological Life Sciences (Salem, MA, USA). ^13^C-labeled substrates were purchased from Cambridge Isotope Laboratories (Tewkbury, MA, USA). Glucose enzymatic kits were purchased from Boehringer Mannheim. All other chemicals and enzymatic kits were purchased from Sigma-Aldrich (St. Louis, MO, USA). The *Y. lipolytica* strain CLIB138 (*MatB, leu2*-*35, lys5*-*12, ura3*-*18, xpr2LYS5*) was purchased from CIRM-Levures (Thiverval-grignon, France) and used as host cells in the study. *Y. lipolytica* was grown in YPD medium (per liter: 10 g yeast extract, 20 g peptone, 20 g of glucose) or YNB medium (per liter: 6.7 g yeast nitrogen base with ammonia sulfate, 1.72 g SD drop-out supplemented with 10 or 20 g of glucose). Strains were maintained on YPD agar plates (YPD medium supplemented with 20 g/L of agar). All growth conditions followed a similar procedure. A single colony (from fresh plates) was used to inoculate seed cultures in the YPD medium (containing 10 g/L glucose) and grown overnight at ~ 30 °C in shaking flasks (250 rpm). The seed culture was used for inoculation of sub-cultures (initial OD_600_ of ~ 0.03). For β-ionone strain cultures, a dodecane layer (10% by volume) was employed for product trapping. Growth was monitored by measuring OD_600_ using a UV–vis spectrophotometer. Glucose and glycerol concentrations were measured using enzymatic kits.

### Plasmid construction for chromosome integration

The primers used to construct *Y. lipolytica* expression vectors are listed in Table 1. The orotidine 5′-phosphate decarboxylase (*URA3*) containing the LoxP site was obtained by PCR amplification with primers URA3-1 and URA3-2 using *Y. lipolytica* genomic DNA as template. The resulting URA3 fragment was cloned into the pUC57 vector to generate the YAL-URA3 construct. A TEF promoter-XPR2 terminator cassette was constructed by stitching two nucleic acid fragments by PCR amplification. First, a 406 bp nucleic acid fragment comprising the TEF promoter was amplified with the primers TEF-1 and TEF_XPR2-1. A 134 bp nucleic acid fragment comprising the XPR2 terminator was amplified with the primers TEF_XPR2-2 and XPR2-1. The TEF-XPR2 cassette was obtained with primers TEF-2 and XPR2-2. The cassette was then cloned into the YAL-URA3 vector to generate the YAL-URA3-TEF-XPR2 vector. A 572 bp nucleic acid fragment comprising the recombination site rDNA1 and a 822 bp nucleic acid fragment comprising the recombination site rDNA2 were amplified using primers rDNA_1 and rDNA_2, and rDNA_3 and rDNA_4, respectively, using *Y. lipolytica* genomic DNA as a template. The nucleic acid fragments comprising rDNA1 and rDNA2 were cloned into the YAL-URA3-TEF-XPR2 construct to yield YAL-rDNA-URA3-TEF-XPR2.

**Table 1:** List of primers used in the study

Primer	Gene/fragment information	Sequence in 5′ to 3′ orientation
URA3_1	Selection marker	TCC ATA TGA ATT ATG CAT GCA TAA CTT CGT ATA ATG TAT GCT ATA CGA AGT TAT ACC AAA ATG CCC TCC TAC GAA GCT CGA GC
URA3_2	Selection marker	CCA CAT GTG GGA ATT CAT AAC TTC GTA TAG CAT ACA TTA TAC GAA GTT ATC GAG AAA CAC AAC AAC ATG CCC CAT TGG AC
TEF_1	TEF promoter-XPR2 terminator cassette	GGA ATT CCG GGT TTA AAC AGA GAC CGG GTT GGC GGC GTA TTT G
TEF_XPR2_1	TEF promoter-XPR2 terminator cassette	TAG GGT ACC TCT AGA CGT CCA CCC GGG AAG GAT CCT TTG AAT GAT TCT TAT ACT CAG AAG
TEF_XPR2_2	TEF promoter-XPR2 terminator cassette	CTT CTG AGT ATA AGA ATC ATT CAA AGG ATC CTT CCC GGG TGG ACG TCT AGA GGT ACC CTA
XPR2_1	TEF promoter-XPR2 terminator cassette	CCA CAT GTG GAC GTC GAC GCC ACC TAC AAG CCA GAT TTT CTA TTT AC
ARS_1	*Yarrowia* automatously replicating sequence	TCC ATA TGC CAG TCT ACA CTG ATT AAT TTT CGG G
ARS_2	*Yarrowia* automatously replicating sequence	TTG CAT GCA TAA GCT AAA AGT AAC TCG CAG CGC A
rDNA_1	Ribosomal DNA integration site	TCC ATA TGG CGG CCG CGG GTC CGG CTG CCA GTT GCC CAG CCG CCA G
rDNA_2	Ribosomal DNA integration site	ATG CAT GCT GGT GGT AGT AGC AAA TAT TCA AAT G
rDNA_3	Ribosomal DNA integration site	GCG TCG ACG TTG GCG CGC CTG CTT CGG TAT GAT AGG AAG AGC CG
rDNA_4	Ribosomal DNA integration site	CCA CAT GTG CGG CCG CGG CAG ACA CTG CGT CGC TCC GTC CAC
TEF_Casset_1	TEF promoter	GCG TCG ACA GAG ACC GGG TTG GCG GCG TAT TTG
TEF_Casset_2	TEF promoter	TTG GCG CGC CAG AGA CCG GGT TGG CGG CGT ATT TG
XPR2-Casset_1	XPR2 terminator	TTG GCG CGC CGC CAC CTA CAA GCC AGA TTT TCT ATT TAC
ACAT_F:	Acetyl-CoA thiolase	GGA TCC ATG CGA CTC ACT CTG CCC CGA CTT
ACAT_R	Acetyl-CoA thiolase	CCT AGG CTA CTC GAC AGA AGA GAC CTT CTT G
NphT7_F	Acetoacetyl CoA synthase	GGA TCC ATG ACT GAT GTC CGA TTC CGC ATT ATC
NphT7_R	Acetoacetyl CoA synthase	CCT AGG TTA CCA CTC AAT CAG AGC GAA GCT
HMGS_F	HMG-CoA synthase	GGA TCC ATG TCG CAA CCC CAG AAC GTT GG
HMGS_R	HMG-CoA synthase	CCT AGG CTA CTG CTT GAT CTC GTA CTT TCG
IPI_F	IPP isomerase	TGA TCA ATG CTT CGT TCG TTG CTC AGA GGC
IPI_R	IPP isomerase	CCT AGG TCA CGC TTC GTT GAT GTG ATG CAC
tHMGR_F	Truncated HMG-CoA reductase	TGA TCA ATG CGA GAA GTT GTG CGA ACC CAG
tHMGR-R	Truncated HMG-CoA reductase	CCT AGG CTA TGA CCG TAT GCA AAT ATT CGA AC
MK_F	Mevalonate kinase	TGA TCA ATG GAC TAC ATC ATT TCG GCG C
MK_R	Mevalonate kinase	CCT AGG CTA ATG GGT CCA GGG ACC GAT
PMK_F	Phosphomevalonate kinase	GGA TCC ATG ACC ACC TAT TCG GCT CCG GG
PMK_R	Phosphomevalonate kinase	CCT AGG CTA CTT GAA CCC CTT CTC GAG CC
MPD_F	Mevalonate pyrophosphate decarboxylase	TGA TCA ATG ATC CAC CAG GCC TCC ACC ACC
MPD_R	Mevalonate pyrophosphate decarboxylase	CCT AGG CTA CTT GCT GTT CTT CAG AGA ACC
FPPS_F	Fusion gene of farnesyl pyrophosphate synthase and geranyl pyrophosphate synthase	GGA TCC ATG CGG GAT CCA TGT CCA AGG CGA
FPPS::GGPPS_R	Fusion gene of farnesyl pyrophosphate synthase and geranyl pyrophosphate synthase	AAA TCC GCG CTG TTA TAA TCC ATA GAA CCA CCA CCC TTC TGT CGC TTG TAA ATC TTG G
FPPS::GGPPS_F	Fusion gene of farnesyl pyrophosphate synthase and geranyl pyrophosphate synthase	CCA AGA TTT ACA AGC GAC AGA AGG GTG GTG GTT CTA TGG ATT ATA ACA GCG CGG ATT T
GGPPS_R	Fusion gene of farnesyl pyrophosphate synthase and geranyl pyrophosphate synthase	C CTA GGT CAC TGC GCA TCC TCA AAG TAC TTT C
OfCCD1_F	Carotenoid cleavage dioxygenase 1	GGA TCC ATG GGT ATG CAG GGC GAG GAT GCT
OfCCD1_R	Carotenoid cleavage dioxygenase 1	CCT AGG TTA CAC CTT AGC CTG CTC CTG GAG C

The pathway of β-carotene biosynthesis was reconstructed in *Y. lipolytica* by expressing two enzymes: phytoene dehydrogenase (carB) and bifunctional lycopene cyclase/phytoene synthase (carRP) of *M. circinelloides*. An additional gene encoding the carotenoid cleavage dioxygenase (CCD1) of *O. fragrans* is required for β-ionone synthesis. These three genes were synthesized based on *Y. lipolytica*-preferred codon usage and cloned into YAL-rDNA-URA3-TEF-XPR2 to form the plasmids YAL-rDNA-URA3-TEF-carB, YAL-rDNA-URA3-TEF-carRP, and YAL-rDNA-URA3-TEF-OfCCD1, respectively. The TEF-carRP-XPR2 and TEF-OfCCD1-XPR2 cassettes were obtained by PCR amplification with primers TEF_Casset_1 and XPR2-Casset_1 and TEF_Casset_2 and XPR2-Casset_1, respectively. The amplified *carRP* expression cassette was cloned into YAL-rDNA-URA3-TEF-carB to generate YAL-rDNA-URA3-TEF-carB-carRP for β-carotene synthesis and YAL-rDNA-URA3-TEF-carB-carRP-OfCCD1 for β-ionone synthesis. In order to enhance precursor supply, eight native genes were over-expressed and two heterologous genes were expressed. Each plasmid contained two gene expression cassettes [(1) Acetyl-CoA thiolase (*ACAT*) and Acetoacetyl CoA synthase (*NphT7*) from *Streptomyces*; (2) HMG-CoA synthase (*HMGS*) and Truncated HMG-CoA reductase (*tHMGR*); (3) Mevalonate kinase (*MK*) and Phosphomevalonate kinase (*PMK*); (4) Mevalonate pyrophosphate decarboxylase (*MPD*) and IPP isomerase (*IPI*) from *Haemotoccus pluvialis*); (5) and a fusion gene of farnesyl pyrophosphate synthase (*FPPS*) and geranyl pyrophosphate synthase (*GGPPS*)]. The plasmids targeted the rDNA sites for integration into the chromosome, which has previously been shown to result in multiple gene copies [48, 49]. Native genes were obtained via PCR amplification using *Y. lipolytica* genomic DNA as template. Exogenous genes were codon-optimized. As such, all overexpressed genes had the same promoter and terminator (the TEF promoter and XPR2 terminator) [29, 33–35]. The Cre-LoxP system was employed to remove the selection markers after each round of genome integration. The *URA3* fragment of YAL-URA3-TEF-XPR2 vector was replaced by the *LEU2* fragment to yield the YAL-LEU2-TEF-XPR2. The *Y. lipolytica* autonomously replicating sequence (ARS) was amplified using primers ARS_1 and ARS_2, and cloned into the YAL-LEU2-TEF-XPR2 vector, to generate vector YAL-LEU2-TEF-XPR2-ARS. The Cre recombinase gene (*Cre*) was synthesized, and cloned into the YAL-LEU2-TEF-XPR2-ARS vector, to yield the YAL-LEU2-Cre vector for marker excision.

### Transformation and marker excision

For β-carotene synthesis, the *Y. lipolytica* strain CLIB138 was transformed with linear plasmid YAL-rDNA-URA3-TEF-carB-carRP by *Not*I on SD-Uracil selection plates according to the Frozen EZ Yeast Transformation Kit II (Zymo Research, CA). The positive colonies were identified via color screening and HPLC analysis. The strain was subsequently transformed with YAL-LEU-Cre for URA3 marker excision as describe in Fickers et al. [50]. The resulting strain without the marker selection gene was used for the next round of transformations. To enhance GGPP supply, ten genes were transformed into the β-carotene producing strain via five rounds of transformations and marker rescue. The Ura+ transformants were identified by PCR with each gene specific primer and TEF promoter primer. The resulting engineered strain was transformed with the linearized plasmid YAL-rDNA-URA3-TEF-carB-carRP-ofCCD1 for β-ionone synthesis, and to incorporate additional copies of *carB* and *carRP* to possibly increase β-carotene accumulation.

### Tests for nutrient effects on the β-carotene production

Shaking tube cultures (5 mL) were used to screen nutrient effects. YNB medium was supplemented with combinations of 2 g/L of a secondary carbon source (acetate, ethanol, glucose, glycerol, pyruvate) or 0.5 g/L of an amino acid (isoleucine, leucine, methionine, phenylalanine, valine) added at either 0, 18 or 24 h (see Additional file 1). The resulting β-carotene titers and yields per biomass were normalized to the control (control conditions: cells grown in YNB medium supplemented with an additional 2 g/L glucose) and analyzed by a machine learning model. Based on model predictions, the defined medium YNB(+) was formulated by augmenting YNB medium with 2 g/L glycerol, 0.25 g/L valine and 0.25 g/L isoleucine (total of 0.5 g/L amino acid), and with 0.5 g/L ethanol added during the middle-log phase. An Eppendorf BioFlo 120 Bioprocess Control Station (2 L working volume) was used for β-carotene fermentation (pH 5.5 controlled by 30% NaOH; dissolved oxygen minimum set at 20%, with 100% as the initial DO, 0% baseline noise). The β-carotene YPD medium fermentation was fed-batch (250 mL 10× YPD medium followed by a total of ~ 225 g glucose, batch added in equal aliquots at hours 72, 96, and 120).

### Machine learning of shaking tube data

Machine learning was performed in the Anaconda JupyterLab Python environment with the Scikit-learn packaging [51]. Data was preprocessed to form two categories: a high performance group (top 10% β-carotene production) and a ‘background’ group. A Gradient Boosting Classifier with 25 trees and five features was trained on 70% of the data. The remaining 30% of data was used to predict the accuracy of the model. All the accuracy metrics and feature importance scores reported were based on permutation method to avoid sampling biases. To better evaluate the effect of each feature on predicting, we implemented a procedure which is similar to the one described in Azure Machine Learning Studio to evaluate feature importance. The fitting procedure was described as below:

- Step 1: We randomly split the data into a training set and a test set (70% for training).
- Step 2: Trained a Gradient Boosting Classifier with 25 trees and five features on the training data set (the accuracy of this model on the test data set is reported).
- Step 3: For feature importance, we randomly shuffled the first feature while keeping the other features unchanged, recalculated the model prediction (built in Step 2) on the shuffled test data, and recorded the difference in prediction accuracy between the original test set and shuffled test set. The difference is recorded as the importance score for the first feature in this round. We sequentially implemented this procedure for all features and obtained their importance scores.
- Step 4: Repeated Step 1 to Step 3 for 1000 times.
- Step 5: Averaged the accuracy and feature importance scores recorded in each round.

The python code for these will be available upon request.

### β-Ionone fermentations

For β-ionone fermentation, 25 mL YPD medium seed cultures were used to inoculate an Eppendorf BioFlo 120 Bioprocess Control Station (2 L working volume) containing 850 mL of YPD medium (initial OD ~ 0.7). 150 mL of dodecane was used as the initial organic overlay layer and was added in a batch manner to maintain an approximately 10% dodecane layer. During 6 day fermentation period, 250 mL 10× YPD was gradually added to promoted biomass growth during day 1 followed by feeding 500 g/L glucose at rate of 0.2 mL/min (28–30 °C, pH 5.50, DO ~ 20%). During fermentation, time-course samples were taken to monitor cell growth and ionone production. In other tests, the fed-batch fermentations were run by continuously feeding cell with 50% glycerol (instead of glucose) with the addition of extra valine (0.25 g/L) and isoleucine (0.25 g/L) in the medium. Finally, we tested strain production stability. Specifically, the engineered β-carotene and β-ionone strains were plated on YPD agar from their respective glycerol stocks. Then a single colony was re-plated. The colonies from second set of plates were used to inoculate 25 mL YPD medium in shaking flasks. After 24–26 h of cultivation, the strains were routinely sub-cultured (starting OD_600_ ~ 0.02) until the loss of production was observed.

### β-Carotene and β-ionone measurements

β-Carotene was measured following a reported method [36]. Briefly, centrifuged biomass pellets were washed and re-suspended in 1 mL of dodecane. 250–400 µg of acid washed glass beads were added and the mixture was vortexed until no orange pellet was observed. 200 µL of dodecane was then transferred to a Corning Inc. 96-well assay plate (black plate, clear bottom with lid). The absorbance was measured on a Tecan Infinite M200 Pro Microplate Reader at 454 nm. A standard curve was used to quantify β-carotene. To confirm β-carotene production, an Alliance 2996 HPLC (Waters, MA) equipped with a 2476 photodiode array detector was employed. Samples were separated by reverse-phase chromatography on a YMC carotenoid column (particle size 5 µm; 250 × 4.6 mm) isocratically using a mobile phase of methyl-t-butyl ether/methanol/ethyl acetate (in a 40:50:10 vol/vol/vol ratio) at a flow rate of 0.5 mL/min for 25 min.

β-Ionone in dodecane layers were directly measured by GC–MS. β-ionone in the aqueous medium and cell pellets were extracted using dodecane before GC–MS analysis. An Agilent Technologies gas chromatography (7820A GC system equipped with a HP-5 column) and a single quadruple mass analyzer (5977E MSD) were used for ionone analyses. Operational conditions were as follows: the GC was initially held at 100 °C for 3 min, and then ramped at a rate of 3 °C/min until 145 °C was reached. The inlet port was held at 250 °C. The MS source was held at 230 °C while the quadruple was at 150 °C. Helium gas was used as a carrier gas. β-Ionone retention times were confirmed using both standards and published mass spectrums [52]. Standard curves were built to quantify the extracted ionone concentration.

### Isotopic labeling and analysis

The seed cultures inoculated a 5 mL YNB medium containing 10 g/L U-^13^C (fully) labeled glucose at a 0.5 vol/vol %. In the early exponential phase, cultures were pulsed with either unlabeled glycerol or ethanol to final concentrations of 5 g/L or isoleucine or valine to final concentrations of 0.5 g/L. Cells were harvested 24 h after the pulse. Free metabolites were analyzed by LC–MS in Joint Bio-Energy Institute. Specifically, cell metabolism was quickly quenched using liquid N_2_ and centrifuged at 5500 rpm for 5 min at 0 °C [53]. LC–MS was operated as previously described [53]. The only differences were that the mobile phase was changed to 20 mM ammonium carbonate (Sigma-Aldrich, St. Louis, MO, USA) in water (solvent A) and 20 mM ammonium carbonate in 70% acetonitrile and 30% water (solvent B); the column compartment was set to 40 °C, and the liquid chromatography gradient was changed as follows: Linearly decreased from 100% B to 70% B in 9 min, decreased from 70% B to 60% B in 2.8 min, increased from 60% B to 100% B in 0.2 min, and held at 100% B for a further 10 min. The total LC run time was 22 min. A flow rate of 0.2 mL/min was used throughout.

## Additional file

**Additional file 1.** Small tube medium test data. This file contains the small tube medium test data labels for Fig. 2 as well as the data input for machine learning analysis.

## Abbreviations

CCD: carotenoid cleavage dioxygenase
DCW: dry cell weight
FPPS: farnesyl pyrophosphate synthase
GGPP: geranylgeranyl pyrophosphate
GGPPS: geranyl pyrophosphate synthase
GPS: geranyl phosphate synthase
GRAS: generally recognized as safe
HMGS: 3-hydroxy-3-methyl-glutaryl-coenzyme A synthase
IPI: isopentenyl/dimethylallyl diphosphate isomerase
IPP: isopentenyl pyrophosphate
MK: Mevalonate kinase
MPD: Mevalonate pyrophosphate decarboxylase
PMK: Phosphomevalonate kinase
rpm: rotations per minute
tHMGR: Truncated HMG-CoA reductase
*Y. lipolytica*: *Yarrowia lipolytica*
YNB: yeast nitrogen base
YPD: yeast extract-peptone-dextrose
